# Magnetic field in the extreme low frequency band protects neuronal and microglia cells from oxygen-glucose deprivation

**DOI:** 10.3389/fncel.2024.1455158

**Published:** 2024-11-01

**Authors:** Paloma Mata, Stefano Calovi, Kami Pars Benli, Leyre Iglesias, María Isabel Hernández, Abraham Martín, Alberto Pérez-Samartín, Ander Ramos-Murguialday, María Domercq, Iñaki Ortego-Isasa

**Affiliations:** ^1^Achucarro Basque Center for Neuroscience, Leioa, Spain; ^2^Department of Neuroscience, University of the Basque Country UPV/EHU, Leioa, Spain; ^3^Semi Zabala, Donostia-San Sebastián, Spain; ^4^Ikerbasque Basque Foundation for Science, Bilbao, Spain; ^5^Centro de Investigación Biomédica en Red de Enfermedades Neurodegenerativas (CIBERNED), Leioa, Spain; ^6^TECNALIA, Basque Research and Technology Alliance (BRTA), San Sebastian, Spain; ^7^Institute of Medical Psychology and Behavioral Neurobiology, University of Tübingen, Tübingen, Germany; ^8^Department of Neurology and Stroke, University of Tubingen, Tubingen, Germany; ^9^Athenea Neuroclinics, Donostia-San Sebastian, Spain

**Keywords:** stroke, extreme low frequency electromagnetic stimulation (ELF-EMS), oxygen and glucose deprivation, cell viability, neuron, microglia

## Abstract

Ischemic stroke consists of rapid neural death as a consequence of brain vessel obstruction, followed by damage to the neighboring tissue known as ischemic penumbra. The cerebral tissue in the core of the lesions becomes irreversibly damaged, however, the ischemic penumbra is potentially recoverable during the initial phases after the stroke. Therefore, there is real need for emerging therapeutic strategies to reduce ischemic damage and its spread to the penumbral region. For this reason, we tested the effect of Extreme Low Frequency Electromagnetic Stimulation (ELF-EMS) on *in vitro* primary neuronal and microglial cultures under oxygen-glucose deprivation (OGD) conditions. ELF-EMS under basal non-OGD conditions did not induce any effect in cell survival. However, ELF-EMS significantly reduced neuronal cell death in OGD conditions and reduced ischemic induced Ca^2+^ overload. Likewise, ELF-EMS modulated microglia activation and OGD-induced microglia cell death. Hence, this study suggests potential benefits in the application of ELF-EMS to limit ischemic irreversible damages under *in vitro* stroke conditions, encouraging *in vivo* preclinical validations of ELF-EMS as a potential therapeutic strategy for ischemic stroke.

## 1 Introduction

Stroke is a leading cause of mortality and sustained disability worldwide (Norrving et al., 2018; Irastorza-Landa et al., 2022, 2023). At the beginning of the 21st century, ~1.1 million inhabitants in Europe suffered a stroke each year with one-month case-fatality rates ranging from 13 to 35% (Chamorro et al., 2016). For this reason, stroke is an unquestionable emergency and a grand challenge for basic and clinical neuroscientists. Despite the remarkable progress in the care of acute stroke with thrombolysis and mechanical thrombectomy during last years (Gauberti et al., 2021), the clinical management of ischemic stroke still remains limited due to the lack of effective treatments (Vidaurre et al., 2023). Therefore, establishing novel neuroprotective strategies will contribute to accelerate the approval of promising neuroprotective therapies that could prevent the progression of the ischemic penumbra to brain infarction. In the ischemic penumbra where damage is potentially reversible, neuronal activity is altered due to ischemia-induced synaptic failure and the early neural activation is hypothesized as a neuroprotective mechanism of great impact in reducing neuroinflammation and cell death (Uzdensky, 2020).

In this sense, electromagnetic stimulation using different electric or magnetic stimulation procedures have shown the potential to neuromodulate the neuronal activity of the ischemic penumbra (Matsuura et al., 2015; Boonzaier et al., 2018; Moya Gómez et al., 2021). In the present study, we have focused on the use of Extreme Low Frequency Electromagnetic Stimulation (ELF-EMS) since has already shown its ability to modulate cellular processes that occur in the ischemic cascade (Moya Gómez et al., 2021). ELF-EMS are usually generated using a solenoid or a collection of coils to create a homogeneous magnetic field on the region of interest. Frequency range is usually in the range of 0 to 100 Hz and magnetic fields from 50 μT to 10 mT are employed (Cheng et al., 2015; Moya Gómez et al., 2021). Several mechanisms of action have been postulated for ELS-EMS following *in vitro* ischemic conditions, from interactions with cellular membranes to alterations in intracellular calcium concentration and reduction in ROS levels (Duong and Kim, 2016). Other effects include the inhibition of apoptosis (Palumbo et al., 2006) or the induction of nitric oxide (NO) production (Akdag et al., 2007; Cichoń et al., 2017b) and angiogenesis. Likewise, different studies have documented the application of ELF-EMS in preclinical rodent models of stroke resulting on the reduction of brain oedema, infarct volume size and inflammation (Grant et al., 1994; Pena-Philippides et al., 2014) and increasing survival (Font et al., 2019). In the last years, clinical results have also showed the great potential of ELF in post-stroke patients (Capone et al., 2017; Cichoń et al., 2017a,b; Cichon et al., 2018, 2019; Cichoń et al., 2018) showing an increase of growth factors and cytokines levels associated with neuroplasticity and functional recovery (Cichoń et al., 2018).

Nevertheless, the wide variety of ELF-EMS protocols (varying in terms of intensity, frequency, waveform, and duration), together with the diversity of *in-vitro*, preclinical (animal models) and clinical studies, makes it difficult to compare results between studies. In addition, the research of ELF-EMS applied to cerebral ischemia is relatively new and additional studies are needed to better understand the physiological mechanisms involved. In addition, the effect of ELF-EMS on important pathophysiological mechanisms such as the role of Ca^2+^ overload in stroke (Moya Gómez et al., 2021) has generated some discrepancies so far. For this reason, this work has evaluated the protective effect of homogeneous ELF-EMS at 50 Hz (using a sinusoidal waveform) with a magnetic field of 1 mT on cellular death and inflammation following *in vitro* ischemia in primary neuronal and microglial cultures.

## 2 Materials and methods

### 2.1 Animals

All experiments were performed in Sprague Dawley rats according to the procedures approved by the Ethics Committee of the University of the Basque Country (UPV/EHU, M20/2022/416). Animals were handled in accordance with the European Communities Council Directive. Animals were kept under conventional housing conditions (22 ± 2°C, 55 ± 10% humidity, 12-h day/night cycle and with ad libitum access to food and water) at the University of the Basque Country animal unit. All possible efforts were made to minimize animal suffering and the number of animals used.

### 2.2 Cell cultures

#### 2.2.1 Neurons

Primary neuronal cultures were obtained from the neocortex of rat embryos on embryonic day 18 (E18). After brain extraction and homogenization, the cells were suspended in supplemented neurobasal^®^ medium (with B27 supplement, 2 mM glutamine, and antibiotic/antimycotic) and 10% FBS for the initial 24 h *in vitro*. On the second day *in vitro* (2 DIV), the cells were switched to supplemented neurobasal^®^ medium containing 0.2% gentamicin. The cells were seeded in 48-well plates at a density of 100,000 cells per well, which were pre-treated with poly-L-lysine (0.01 mg/ml). As previously determined, at least 98% of the cells were neurons, as determined by immunolabeling with a monoclonal anti-microtubule associated protein antibody (MAP2, Sigma), and the majority of remaining cells were GFAP+ (Ibarretxe et al., 2006). After allowing the neurons to mature for 10 DIV, the ELF-EMS experiments were performed.

#### 2.2.2 Microglia

Primary mixed glial cultures were prepared from the cerebral cortex of neonatal rats (P0-P2). After 10–15 days in culture, microglia were isolated by mechanical shaking (400 rpm, 1 h) and purified by plating on non-coated bacterial grade Petri dishes (Sterilin; Thermo Fisher) as previously described (Domercq et al., 2007). Microglial cells obtained with this procedure were cultured in Dulbecco's Modified Eagle Medium (DMEM; Gibco) supplemented with 10% Fetal Bovine Serum (FBS; Gibco). The ELF-EMS experiments were conducted when the cells reached 2 DIV.

### 2.3 Electromagnetic field exposure system and simulations

**Figure 1:**
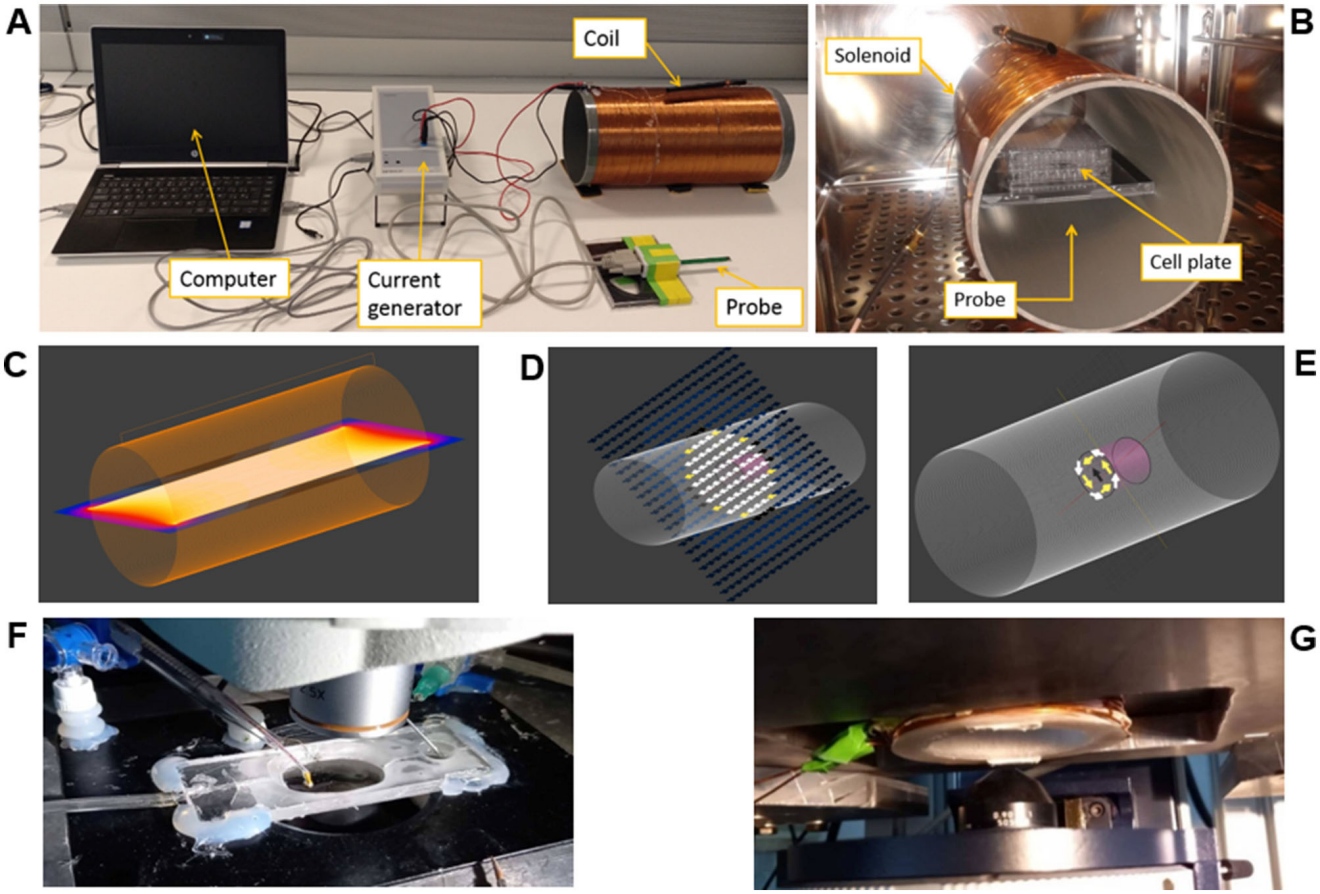
Magnetic stimulation and monitorization setup. **(A)** set-up showing all the components (computer, current generator, coils and the probe to measure the magnetic field inside the solenoid, **(B)** the stimulation (coil) and monitorization (probe) system inside the incubator. **(C, D)** Simulation using Sim4Life of the magnetic field distribution with *B* = 1 mT and *f* = 50 Hz inside the solenoid. **(E)** Induced electric field. **(F)** Top view of the perfusion chamber and microscope components. **(G)** Bottom view of the stage, showing the stimulation coil.

The ELF-EMS system is based on a home-made solenoid, a commercial current generator (Power Cassy) and magnetic probe (Axial B sensor S, ±1,000 mT), both from LD Didactic (Figure 1A). This system is introduced into the CO_2_ incubator, as shown in Figure 1B, where the cell plate is placed in the middle of the solenoid.

Firstly, the analytical Equation 1 has been used to estimate the number of turns, intensity and length needed for the solenoid that will generate the desired magnetic field.


(1) $$\begin{array}{l} B=\;\frac{\mu_{0}IN}{L} \end{array}$$

where, μ_0_ is the permeability constant with a value of 1.26 × 10^−6^ T/m, *N* is the number of turns in the solenoid, *I* is the current passing through the coil, *L* is the coil length.

A solenoid of *N* = 350, *L* = 35 cm and *r* = 80 mm has been designed, modeled and simulated using SIM4LIFE Finite Element Method (FEM) software created by Zurich Medtech (ZMT) (Sim4Life, 2024). A magnetic field of *B* = 1 mT at *f* = 50 Hz (sinusoidal waveform) has been used. The obtained magnetic field distribution is uniform in the middle of the solenoid (Figure 1C). The magneto quasi-static low-frequency solver has been used to calculate the magneto-quasi-static vector potential using the Bio-Savart equation. The induced electric field estimation in the cell plate has been also calculated (Figures 1D, E). The magnetic field was monitored with the probe before and during the study.

A specific coil (Figures 1F, G) was designed and fabricated to be adapted to the microscope (following the same methodology) to allow intracellular calcium monitoring while ELF-EMS was applied (see Section 2.5).

### 2.4 Experimental protocol

In all experiments, a comparison between two plates derived from the same cell culture has been done to investigate the impact of the magnetic field within a physiological context. One plate was incubated under standard conditions, while the other plate was placed in the same incubator but inside the designed solenoid for 24 h.

The plate receiving ELF-EMS was always placed in the central part of the coil to ensure a homogeneous stimulus throughout the 24-h period, as shown in Figure 1B.

### 2.5 Oxygen and glucose deprivation (OGD)

*In-vitro* ischemia (1 h) was achieved by replacing O_2_ with N_2_ and external glucose (10 mM) with sucrose and adding iodoacetate (20 μM, 50 μM) to block glycolysis in an extracellular solution containing (in mM) NaCl (130), KCl (5.4), CaCl_2_ (1.8), NaHCO_3_ (26), MgCl_2_ (0.8), and NaH_2_PO_4_ (1.18) (pH 7.4). Ischemia was induced in the presence of iodoacetate to prevent metabolism of retained intracellular glucose as previously described (Leary et al., 2002; Garnier et al., 2003; Domercq et al., 2010).

### 2.6 Cell death assays

Cell death was determined 24 h later after OGD. To quantify neuronal and microglial viability, these were loaded with 1 M calcein-AM (C3100MP; Invitrogen) and fluorescence was measured using a Synergy H4 Hybrid Multi-Mode Fluorimeter (BioTek Instruments). Excitation and emission wavelengths were as suggested by the supplier. The total number of surviving cells on each well emitting calcein fluorescence was quantified and results were expressed as percentage of surviving cells vs. control. Results were expressed as the mean ± SEM of at least three independent experiments performed in triplicate or quadruplicate. In addition to calcein, neuronal cultures were labeled with the fluorescent nuclear dye Nuc Blue (R37605; Invitrogen) to quantify total number of cells.

### 2.7 Intracellular calcium imaging

For Ca^2+^ recording, cells were loaded with fura-2M (5 μM; Molecular Probes, Eugene, OR) in culture medium for 30 min at 37°C. Experiments were carried out in a coverslip chamber continuously perfused with a buffer containing 20 mM HEPES, 2 mM CaCl_2_, 10 mM glucose in HBSS at 1 ml/min. Recordings were conducted using a Polychrome V monochromator (Till Photonics, Germany) and a Leica DMFSLA microscope (Leica, Germany) equipped with a BP 505 nm suppression filter, a 40 × water immersion objective, and a 10 × eyepiece in the optical path. Images following excitation at 340 and 380 nm were sequentially captured with a high-sensitivity ORCA C9100 camera (Hamamatsu, Japan; exposure time: 21 ms, binning 2 × 2, 1 image/15 seconds), digitized, and processed to calculate the intensity ratio using the radiometric module of Aquacosmos software (Hamamatsu, Japan). To simulate ischemia we replaced external O_2_ by N_2_, and external glucose by sucrose, added 1 mM iodoacetate to block glycolysis and 2.5 μM antimycin to inhibit oxidative phosphorylation, as previously described (Káradóttir et al., 2005) with modifications (Domercq et al., 2010). Without iodoacetate and rotenone/antimycin, it took ~5-fold longer for the ischaemia-evoked depolarization to develop, probably because in an open chamber O_2_ can diffuse to the slice, allowing mitochondrial metabolism to persist longer than it would in cultured cells within the incubator. ELF-EMS was achieved using a coil positioned beneath the recording chamber to allow the magnetic field lines to pass through the sample with minimal attenuation. Before the series of experiments, the system was calibrated to achieve a 1 mT level at the center of the chamber (Figures 1F, G). Analysis was performed in three different cultures and in each culture at least two coverslips and at least 9–10 cells for each coverslip were analyzed.

### 2.8 Immunocytochemistry

Cells were fixed in 4% p-formaldehyde (PFA) in PBS and processed for ICC as previously described (Zabala et al., 2018). Primary antibodies were used as follows to: iNOS (1:500, BD Bioscience, #610329), MRC1 (1:1,000, Abcam #64693) and Iba1 (1:500, Wako #019-19741). As secondary antibodies, goat anti-rabbit Alexa Fluor 488 (1:250, Invitrogen #A11008), goat anti-rabbit Alexa Fluor 594 (1:250, Invitrogen #A21135), goat anti-mouse Alexa Fluor 594 (1:250, Invitrogen #A11012) and goat anti-rat Alexa Fluor 488 (1:250, Invitrogen) were used. Cells were blocked with 4% normal goat serum in PBS containing 0.1% Triton-X100 (blocking solution) and then incubated overnight at 4°C with primary antibodies diluted in blocking solution. Staining was revealed with appropriate secondary antibodies conjugated with Alexa 488 or 594 and incubated for 1 h at room temperature. Images were acquired using a laser scanning confocal Olympus Fluoview FW500 microscopy or a Leica TCS STED CW SP8 super resolution microscope, using the same settings for all samples within one experimental group.

All the image analysis was performed with the ImageJ software (NIH). Morphology analysis of microglia was performed with ImageJ software as described before (Fontainhas et al., 2011). The area of the cell as well as the cell circularity was determined on regions of interest (ROIs) manually selected on the basis of Iba1 immunostaining using the smooth polygon tool NIH ImageJ. Both parameters were analyzed in individual cells in 30–50 cells in three independent experiments. The expression levels of iNOS and MRC1 were analyzed at the single-cell level. Mean gray value (fluorescence intensity/cell) of iNOS and MNR labeling was calculated in individual cells (data was obtained from 20 to 30 cells per coverslip from three different experiments performed in duplicate).

### 2.9 Cytokine measurement

The amount of IL-1b and IL-4 released in the culture medium during reperfusion (24 h) was measured using rat IL-1b (BMS630; Invitrogen) and rat IL-4 (BMS628; Invitrogen) ELISA kits using the standard at 0–100 pg/ml, according to the instructions of the manufacturer. The absorbance was read at 450 nm in a CLARIOstar Plus microplate reader (BMG LABTECH). Data was obtained from four different experiments performed in duplicate.

### 2.10 Statistical analysis

The statistical analysis of the effects of *in vitro* ELF-EMS on baseline cell viability has been carried out using the *t*-Student test. The data has been paired with respect to the cell culture to minimize variability in parameters such as cell number among different cultures. The statistical analysis of more than two groups were performed with *two-way* ANOVA and Sidak's multiple comparisons test.

## 3 Results

### 3.1 ELF-EMS reduced calcium overload and ischemia induced neuronal cell death

**Figure 2:**
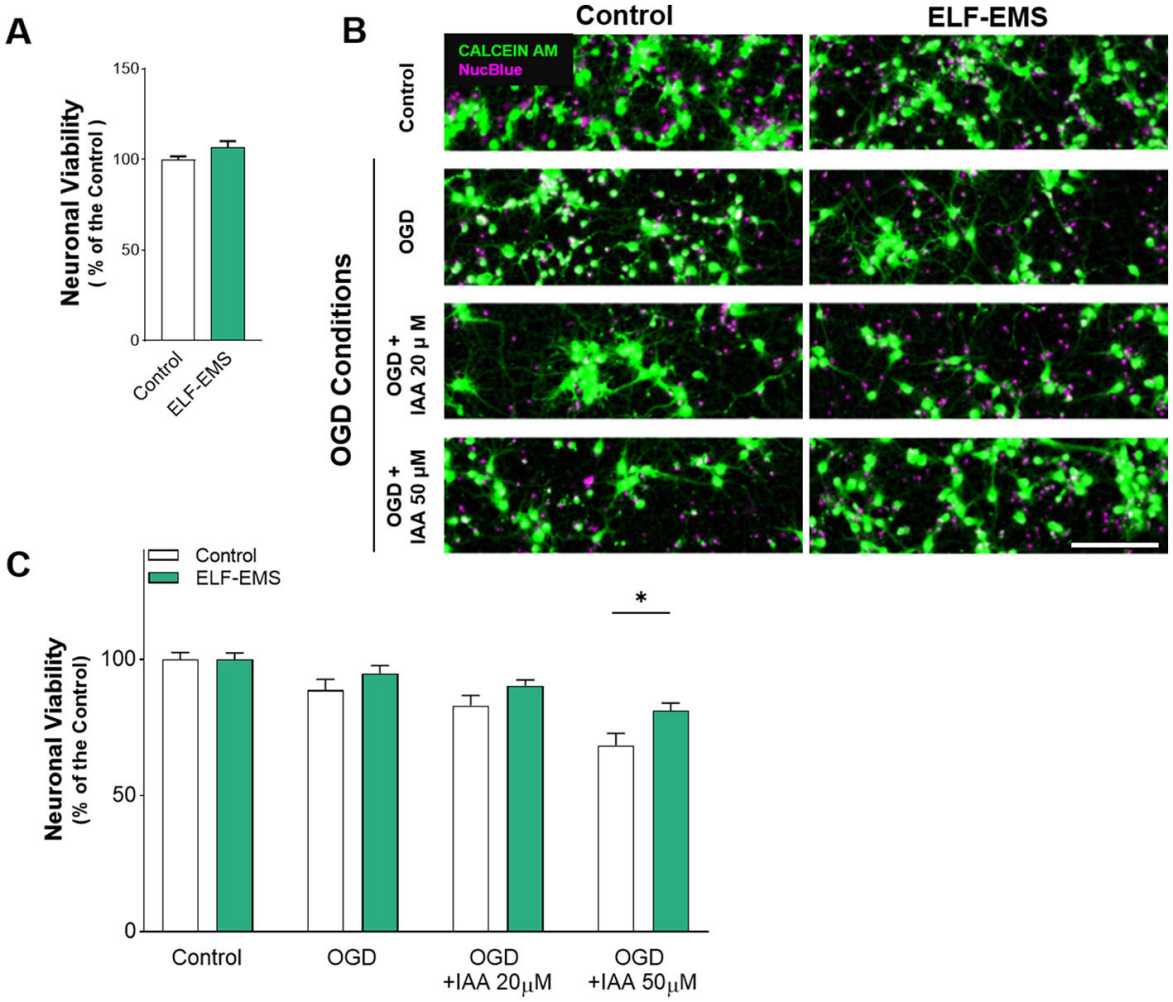
Effect of ELF-EMS (1 mT, 50 Hz; 24 h) on ischemic neuronal death. **(A)** *In vitro* neuronal viability after ELF-EMS (1 mT, 50 Hz; 24 h). Histograms show neuronal viability (mean ± SEM) in percentage vs. the control (*n* = 3 independent experiments performed at least in triplicate). **(B)** Representative images of neuronal cultures *life-*stained with Calcein AM (green) and NucBlue (magenta) in control conditions and after 1 h of OGD in conjunction with iodoacetic acid (IAA)-induced mild (20 μM) or severe (50 μM) chemical ischemia. Scale bar = 100 μm. **(C)** Histograms show neuronal viability (mean ± SEM) after ischemia in the presence or absence of ELF-EMS. Cell death was measured at 24 h of reperfusion; it was reduced by applying ELF-EMS during reperfusion (n= 3 independent experiments performed at least in triplicate). ^*^p < 0.05.

We first optimize the protocol for magnetic stimulation under control conditions. The application of the ELF-EMS (1 mT, 50 Hz) using a solenoid (see Figure 1) for long time incubations (24 h) did not show any effect on neuronal survival under control conditions, as analyzed by calcein-AM fluorometry (Figure 2A). Then, to examine the role of ELF-EMS in ischemia, we simulated energy deprivation by oxygen and glucose deprivation (OGD; 1 h) in the absence or in the presence of the glycolysis blocker iodoacetate (IAA) whereas reperfusion was simulated *in vitro* by returning cells to normal glucose medium and O_2_. Incubation of the cultures in the OGD solution (1 h) resulted in increased cell death, as analyzed 24 h later (Figures 2B, C). Neuronal cell death was increased by blocking glycolysis with IAA (20 and 50 μM), suggesting that neuronal cells *in vitro* switch metabolism to glycolysis for survival. Notably, applying ELF-EMS (1 mT, 50 Hz) in the reperfusion (24 h) reduced OGD-induced neuronal cell death in all the conditions, OGD, OGD + IAA (20 μM; mild ischemia) and OGD + IAA (50 μM; severe ischemia; Figures 2B, C).

**Figure 3:**
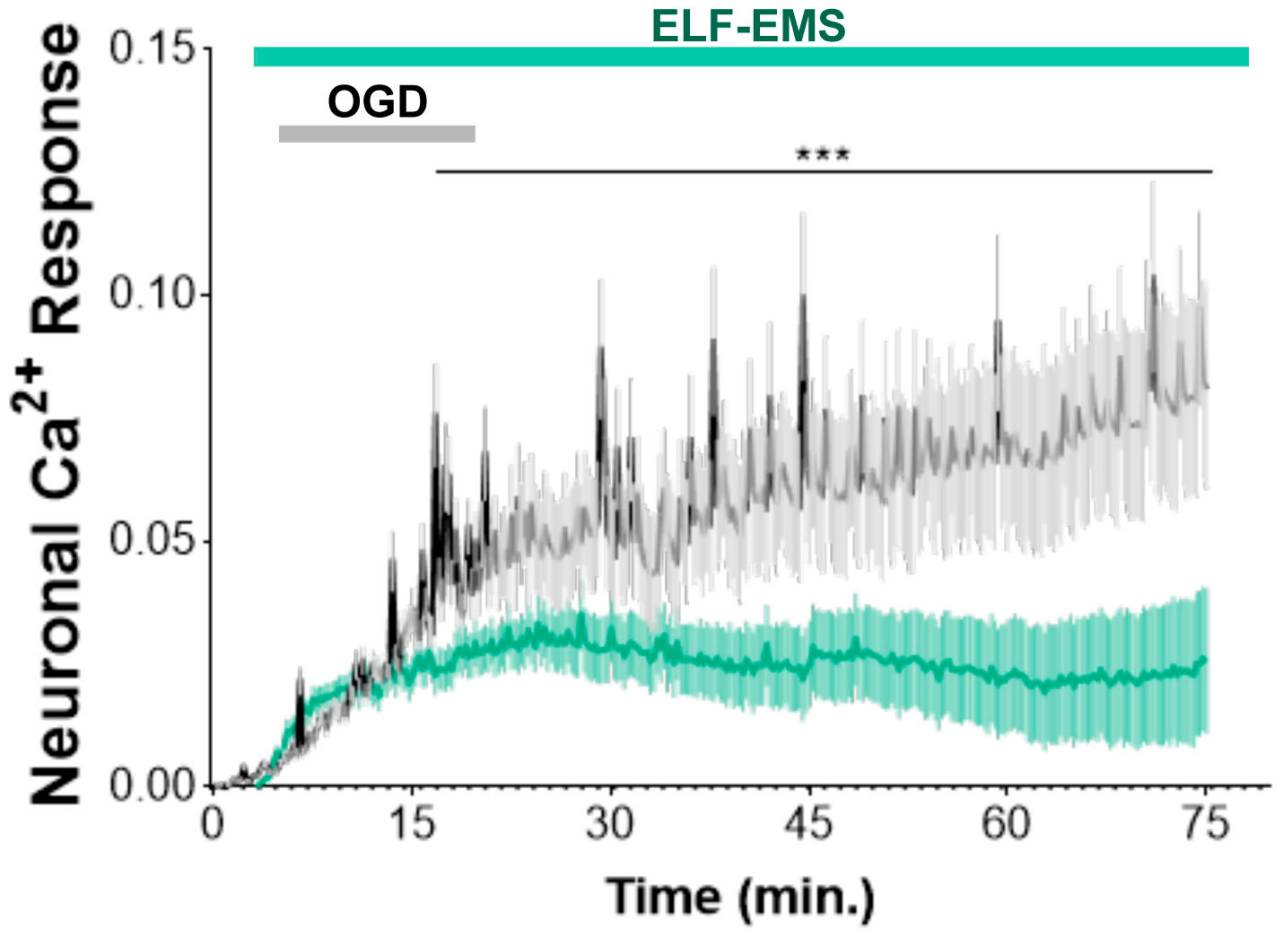
Effect of ELF-EMS (1 mT, 50 Hz) on ischemic Ca^2+^ increase. Averaged traces illustrate the time-course of [Ca^2+^]_i_ increase in neuronal somata after chemical ischemia. The increase was reduced in the presence of ELF-EMS. Graphics represent the mean ± SEM (*n* = 63 cells for control group and *n*= 56 cells for ELF-EMS exposed group coming from three different cultures). ^***^p < 0.001.

Ischemia induced an inward current in neurons called anoxic depolarization (Soria et al., 2014). To simulate and study the anoxic current, we designed a coil positioned beneath the recording chamber that allows us to apply protocols of ELF-EMS and we record by calcium imaging the cytosolic calcium (Figures 1F, G). Chemical ischemia induced a slow increase in cytosolic concentration of Ca^2+^ within minutes (Figure 3), which is attribute to the Ca^2+^ influx through the plasma membrane, as described before (Arbeloa et al., 2012). Importantly, the increase in Ca^2+^ caused by ischemia was significantly reduced by applying ELF-EMS during and after the ischemic period (Figure 3). Thus, our results suggest that ELF-EMS could prevent neuronal cell death by modulating calcium overload secondary to ischemia.

### 3.2 ELF-EMS modulate microglia activation and ischemia induced microglia cell death

**Figure 4:**
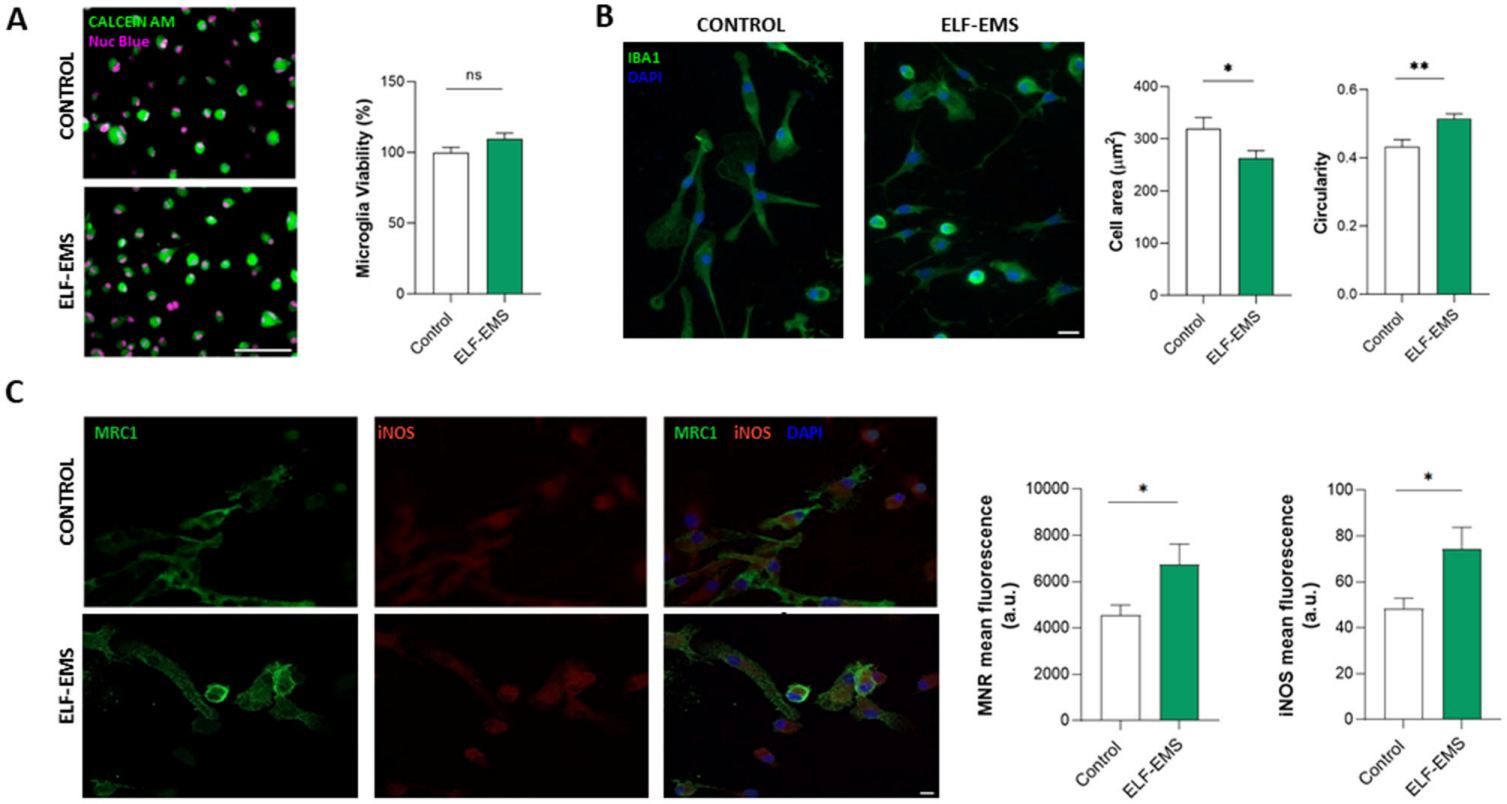
Effect of ELF-EMS (1 mT, 50 Hz; 24 h) on basal microglia activation and viability *in vitro*. **(A)** Representative images of microglia cultures *life-*stained with Calcein AM (green) and NucBlue (magenta). Scale bar = 100 μm. Histograms shows microglia viability (mean ± SEM) in percentage vs. the control condition (*n* = 42 cells for control group and *n* = 54 cells for ELF-EMS group coming from three independent experiments). **(B)** Representative images and morphological analysis of Iba1^+^ microglia. Scale bar = 20 μm. ELF-EMS induced a decrease in microglia area and an increase in microglia circularity (*n* = 33 cells for control group and n= 34 cells for ELF-EMS group from three independent experiments) ^*^p < 0.05. **(C)** Representative images illustrating microglial expression of mannose receptor MRC1 (green), iNOS (red) and DAPI. Scale bar = 20 μm. Histogram shows the quantification of mannose receptor (MRC1, green) and inducible nitric oxide synthase (iNOS, red) expression in microglia cells with or without ELF-EMS (mean ± SEM; *n* = 3 independent experiments performed in triplicate). Scale bar = 20 μm. ^*^p < 0.05. ^**^p < 0.01.

Microglial cells exhibit an extremely plastic response to cellular or tissue damage, which contributes to regeneration and resolution of inflammation, as well as cellular damage (Murray et al., 2014; Xue et al., 2014). This dichotomous nature of microglia has often been associated with the activation of different signaling pathways that can be identified by distinct pro-inflammatory and anti-inflammatory markers (Mosser and Edwards, 2009). As the microglial response is essential for ischemia resolution, we then studied the effect of ELF-EMS in microglia cells. The application of the same ELF-EMS protocols (ELF-EMS; 1 mT, 50 Hz; 24 h) did not cause any effect on microglia survival under control conditions, as analyzed by calcein-AM fluorometry (Figure 4A). However, we observed that ELF-EMS induced significant changes in morphology, acquiring a more ameboid morphology. Indeed, ELF-EMS induced a reduction in microglia area and an increase in cell circularity, an indicative of microglia activation (Figure 4B). In addition, ELF-EMS applied in control conditions induced a significant increase in the expression of both inducible nitric oxide synthase (iNOS), a pro-inflammatory marker, as well as mannose receptor (MRC1), an anti-inflammatory marker (Figure 4C). Thus, although ELF-EMS did not induce any change in microglia viability, it induced an activation of these cells.

**Figure 5:**
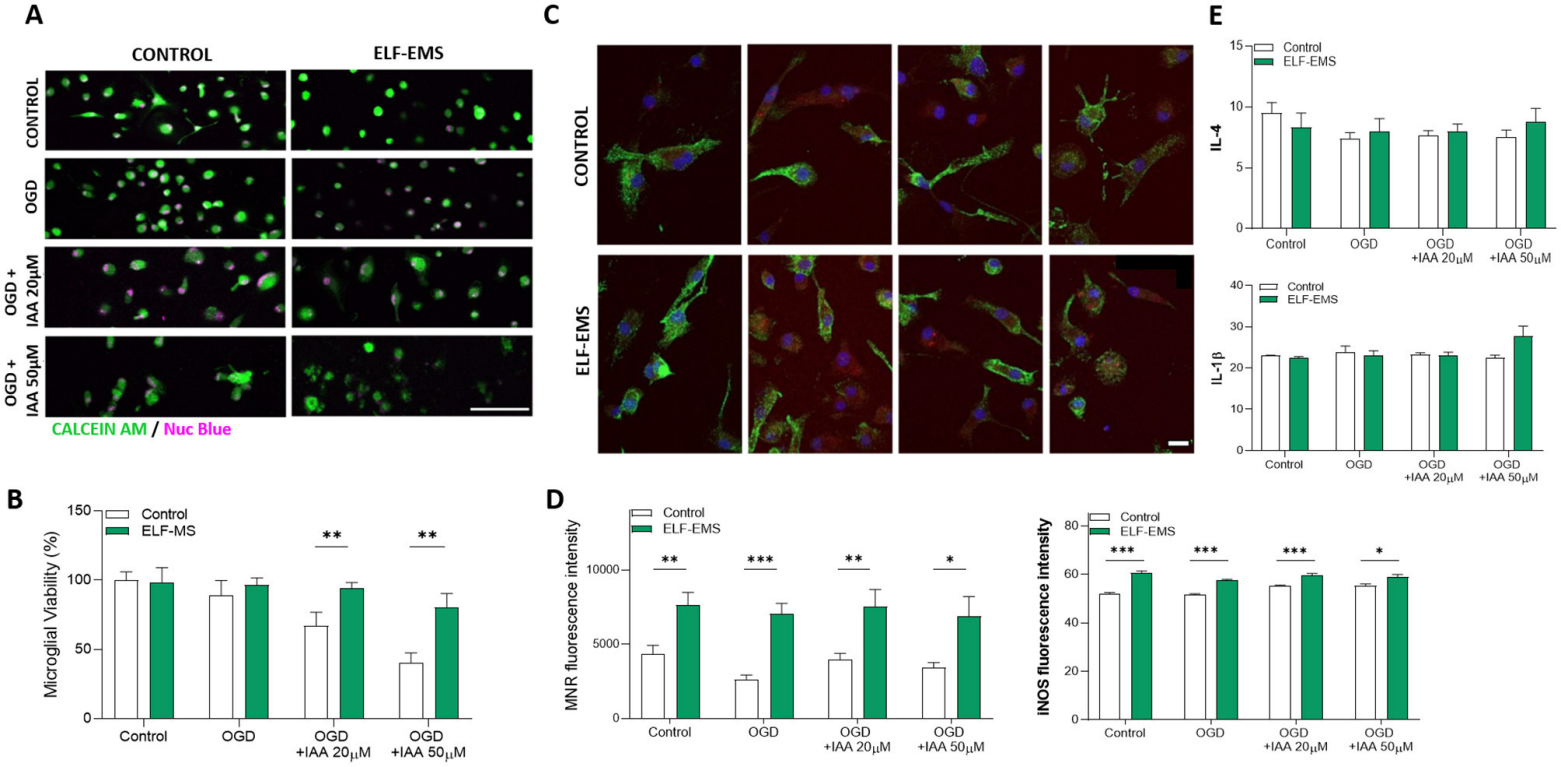
Effect of ELF-EMS (1 mT, 50 Hz; 24 h) on ischemic microglia cell death. **(A)** Representative images of microglial cultures *life-*stained with Calcein AM (green) and NucBlue (magenta) in control conditions and after 1 h of OGD in conjunction with iodoacetic acid (IAA)-induced mild (20 mM) or severe (50 mM) chemical ischemia. Scale bar = 100 μm. **(B)** Histograms show microglial viability (mean ± SEM) after ischemia in the presence or absence of ELF-EMS. Cell death was measured at 24 h of reperfusion; it was reduced by applying ELF-EMS during reperfusion (*n* = 3 independent experiments performed at least in triplicate). ^**^p < 0.01. **(C)** Representative images showing the expression of mannose receptor MRC1 (green), iNOS (red), and DAPI after 1 h *in vitro* OGD. Immunocytochemistry was performed after 24 h of reperfusion. Scale bar = 20 μm. **(D)** Histogram shows the impact of OGD in conjunction with iodoacetic acid (IAA)-induced mild (20 μM) or severe (50 μM) chemical ischemia on MRC1 and iNOS expression (mean ± SEM). ELF-EMS increased both MRC1 as well as iNOS expression (*n* = 3 independent experiments). Scale bar = 20 μm. ^*^p < 0.05; ^**^p < 0.01; ^***^p < 0.005 represent comparison between ELF-EMS stimulated and no stimulated cells. **(E)** Histogram shows the impact of OGD in conjunction with iodoacetic acid (IAA)-induced mild (20 μM) or severe (50 μM) chemical ischemia on IL-1b and IL-4 release, as analyzed by ELISA (mean ± SEM; *n* = 4 independent experiments performed in duplicate).

We then tested the impact of ELF-EMS on cell viability when OGD is applied. Microglia cell death after OGD have been previously described *in vitro* and in acute slices as well as *in vivo* in the ischemic core (Lyons and Kettenmann, 1998; Yenari and Giffard, 2001; Eyo et al., 2013). As in neurons, OGD protocols (1 h) induced an increase in cell death which was exacerbated by blocking glycolysis with iodoacetate (IAA 20 μM and 50 μM; Figures 5A, B). Importantly, applying protocols of ELF-EMS during reperfusion induced a massive reduction of microglia cell death (Figures 5A, B). We then tested the impact of ELF-EMS on microglia cell activation during OGD. We detected a significant increase in the expression of iNOS after OGD + IAA 20 μM (mild ischemia) and after OGD + IAA 50 μM (severe ischemia; Figures 5C, D), but no change in the expression of MCR1. However, in the presence of ELF-EMS, we observed a higher increase in the expression of iNOS and a massive increase in the expression of MRC1 in all the conditions, basal and after OGD conditions (Figures 5C, D). We further quantified the release of pro and anti-inflammatory cytokines IL-1β and IL-4 during reperfusion. Although we did not detect any significant changes, ELF-EMS tends the increase the release of both IL-1β and IL4 in the more severe conditions of ischemia, OGD + IAA 50 μM (Figure 5E).

## 4 Discussion

Previously, ELF-EMS has been applied using different amplitudes (0,1 mT-13.5 mT), frequencies (10–100 Hz), waveform (sinusoidal, pulsed) and time (during hypoxia, after hypoxia at 24 h, 48 h etc.) showing an influence on different biological effects (Moya Gómez et al., 2021). In our study, the stimulations were applied for 24 h after the OGD procedure using an ELF-EMS of 1 mT, 50 Hz and using sinusoidal waveform. Herein, the effect of ELF-EMS was tested on two cell lines, primary neural cultures from the neocortex of rat embryos and primary microglial cultures from the cerebral cortex of postnatal rats.

The biological effects of ELF-EMS are primarily based on the underlying electromagnetic mechanisms, which include the combination of the magnetic field and the induction of microcurrents. A time-varying magnetic field generates an electric field that is proportional to the rate of change of the magnetic field. In our case, the magnetic field of *B* = 1 mT and *f* = 50 Hz generates an electric field on the order of a few mV/m in the cell culture plate. As expected, this value is far below the threshold of the voltage gradient of 100–200 V/m that is required in motor cortex to evoke a muscle response and that can be obtained with commercial transcranial magnetic stimulation (TMS) systems when a short pulse (up to 1 ms) and very high magnetic field (more than 1 T) is applied (Alekseichuk et al., 2019; Ortego-Isasa et al., 2019). It is also below the typical values obtained in in transcutaneous electrical stimulation (tES) techniques such as transcutaneous direct current stimulation (tDCS) or transcutaneous alternated current stimulation (tACS) (Huang et al., 2017; Bland and Sale, 2019) where a typical maximum current of 2 mA creates ~0.8 V/m affecting endogenous network oscillations (Prochaska and Benowitz, 2016; Vöröslakos et al., 2018). To achieve a similar electric field magnitude keeping a frequency of *f* = 50 Hz, we would need to increase the magnetic field by several orders of magnitude. Even considering the significant gap between clinical or pre-clinical scenario and our *in-vitro* approach, the electric field generated with the ELF-EMS is much smaller, making it unlikely that the effects are based on the same mechanisms as those of tDCS or tACS techniques.

Based on this reasoning, we could expect that the magnetic field itself could be responsible of neuromodulation process. So far, it is expected that the magnetic field is transparent for the human body, and consequently should not have a direct effect on CNS cells. However, there are some studies showing promising results using static magnetic fields to modulate different cell mechanisms (Bertolino et al., 2013; Mukhopadhyay et al., 2015; Maredziak et al., 2016). Mukhopadhyay and Paul show a decrease of the infarct size and an increase of brain activity in rats using 40 mT (Mukhopadhyay et al., 2015). This means that maybe, the magnetic field itself is generating a physiological mechanism able to modulate some relevant parameter of the ischemic cascade when OGD is applied without the intervention of an associated electric field.

In this sense there are several *in vitro* and *in vivo* studies that have shown how the ELF-EMS affects different processes of the ischemic cascade (Moya Gómez et al., 2021). Within the ELF-EMS modality, two primary waveforms exist: pulsed and sinusoidal. In our study, we focused on the sinusoidal waveform. Previous studies have described some protective effects of ELF-EMS in mesenchymal stem cells, endothelial cells, as well as human microglia cell line after OGD (Jung and Kim, 2016; Font et al., 2019; Duong and Kim, 2016). However, no study has analyzed the impact of ELF-EMS after OGD in microglia and neuronal primary cultures. Concerning the experimentation with neural cells, the stimulation did not alter cell viability in neuronal cultures, but it significantly reduced cell death in OGD conditions (Figure 2). The stimulation was able to modulate the increase in intracellular Ca^2+^ secondary to OGD (Figure 3). When an ischemic stroke occurs, an ionic perturbation is generated in the membranes of neurons and glia, known as anoxic depolarization, which in turn induces the activation of calcium-dependent channels, leading to massive calcium influx into the cells. Subsequently, these changes lead activation of enzymes responsible for initiating a sequence of cytotoxic events leading to the degradation of proteins, lipids, and nucleic acids (Choi, 1988, 1995). Hence, our results might suggest that the ELF-EMS can provide protection in *in-vitro* ischemia protocols via interfering with mechanisms responsible for anoxic depolarization. Indeed, previous studies using similar stimulation conditions in an immortalized human microglia cell line (HMO6; 50 Hz/1 mT, 4 h simultaneous to hypoxia) (Duong and Kim, 2016) and in mesenchymal stem cells (10 Hz/1 mT, 3 h simultaneous to hypoxia) (Jung and Kim, 2016) showed similar findings. Previous data has also observed a link between ELF-EMS and calcium homeostasis. In myoblasts and myotubes, ELF-EMS was able to increase the spontaneous activity and the intracellular Ca^2+^ (Morabito et al., 2010; Sun et al., 2016). Moreover, ELF-EMS induced an increase in Ca^2+^ channel expression which potentiates short term plasticity at the calyx of Held synapses (Sun et al., 2016). Thus, ELF-EMS could affect calcium dynamics in different cells with consequences in physiological and pathological conditions (Moya Gómez et al., 2021). The role of Ca^2+^ and ELF-EMS remains unclear with controversial results in bibliography due the diversity of stimulation parameters (time, frequency, intensity, waveform) and cell types that can be employed (Moya Gómez et al., 2021).

However, although ELF-EMS seems to be able to modulate calcium overload secondary to ischemia, ELF-EMS protection might be explained by other mechanisms, as ELF-EMS was applied after OGD in the survival experiments. In addition to modulate intracellular Ca^2+^ dynamics, ELF-EMS activates intracellular signal transduction pathways that regulate the balance between neuronal death and survival. It has been shown that ELF-EMS activates the p38 kinase cascade, leading to the recruitment of HSP70, CREB, and BDNF, which are critical for neuronal survival (Moya Gómez et al., 2021). Additionally, the activation of the BDNF/TrkB/Akt pathway by ELF-EMS increases the phosphorylation of Bad, preventing its binding to Bcl-xL (Moya Gómez et al., 2021), thereby reducing apoptosis. These effects are particularly important under conditions of ischemia, as they help to maintain neuronal viability by promoting anti-apoptotic pathways. Morevoer, ELF-EMS reduces oxidative stress, as evidenced by decreased levels of ROS, MMP9, and HIF-1α (Moya Gómez et al., 2021), which are typically elevated during ischemic stress exacerbated by glycolytic inhibition. By reducing ROS production, ELF-EMS helps to preserve ATP production, thereby mitigating the energy deficit caused by OGD plus IAA.

One of the most relevant mechanism of ELF-EMS is the ability to modulate the inflammatory process by microglial activation (Moya Gómez et al., 2021). Microglia are extremely plastic cells, always controlling the brain parenchyma and ready to migrate, proliferate and respond to any type of signal of injury or alteration. Our results indicates that microglia sense the small changes in magnetic field produced by ELF-EMS, as indicated by the change in morphology and in the expression of pro and anti-inflammatory markers (Figures 4, 5). Altough no clear shift between the expression of pro-inflammatory and anti-inflamatory were observed in basal conditions as well as after OGD, a higher upregulation of the anti-inflammatory MRC1 marker was detected in all the conditions tested. These results are in line with recent studies showing beneficial effects of ELF-EMS in a rodent model of global transient stroke by a mechanism involving modulation of microglial migration and the expression of inflammation-related markers (Moya-Gómez et al., 2023). As observed in neurons, ELF-EMS also reduces oxidative stress in microglia by decreasing levels of reactive oxygen species (ROS) (Balind et al., 2014). This reduction in oxidative stress is crucial for preventing microglial cells from ischemic oxidative damage. In addition, ELF-EMS influences the balance of microglial activation. ELF-EMS has been observed to decrease the expression of pro-inflammatory cytokines, such as TNF-α and IL-1β, through the modulation of JNK1/2 pathway (Moya Gómez et al., 2021), contributing to a reduction in microglial-induced inflammation during ischemic events. On the other hand, ELF-EMS upregulates anti-inflammatory cytokines like IL-10, 11, and 13 after stroke (Pena-Philippides et al., 2014).

Overall, these findings are highly relevant in stroke research, as both the anoxic depolarization and the microglia and inflammatory response play an important role in the pathophysiology of ischemic stroke. Moreover, the neuroprotective effects that we have described with neural cells show the great potential of using ELF-EMS as treatment for stroke. Further analyses are needed applying a wide sweep of parameters (frequency, amplitude, time and waveform) to more accurately define different combinations which could help to adapt this technology to a clinical scenario.

## 5 Conclusions

The main objective of this paper is to investigate the potential of ELF-EMS as new treatment for acute ischemic stroke. The ELF-EMS under basal (non-OGD conditions) did not induce any effect in cell survival. However, ELF-EMS significantly reduced cell death in OGD conditions in both neuronal and microglia cell types by modulating the intracellular (Ca^2+^). Moreover, ELF-EMS reduced neuronal anoxic currents, as reported using calcium imaging, and it showed a tendency to activate the expression of both pro- and anti-inflammatory factors on microglia accompanied by an amoeboid morphological shift. Hence, these findings supported the potential of ELF-EMS as a therapeutic strategy to mitigate ischemic damage.

## Data Availability

The raw data supporting the conclusions of this article will be made available by the authors, without undue reservation.
